# Transcriptomic Analysis Provides Molecular Insights into Skin Development in Dezhou Donkey Foals

**DOI:** 10.3390/vetsci13010107

**Published:** 2026-01-21

**Authors:** Tong Li, Honglei Qu, Liyuan Wang, Qiugang Ma, Changfa Wang, Muhammad Zahoor Khan, Wenqiong Chai

**Affiliations:** 1 College of Agriculture and Biology, Liaocheng University, Liaocheng 252000, China; 2 State Key Laboratory of Animal Nutrition, College of Animal Science and Technology, China Agricultural University, Beijing 100193, China; 3 Shandong Key Laboratory of Gelatine Medicines Research and Development, Dong’e Ejiao Co., Ltd., Liaocheng 252201, China

**Keywords:** Dezhou donkey, skin, transcriptome, differentially expressed genes, RNA-seq

## Abstract

Skin development undergoes significant molecular changes during early life stages in mammals. This study investigated transcriptomic differences in skin tissues between newborn (Y0) and one-year-old (Y1) Dezhou donkey foals using RNA-sequencing technology. Skin samples were collected from 13 Dezhou donkeys (7 newborns and 6 one-year-olds) and subjected to transcriptome analysis using the Illumina NovaSeq 6000 platform. A total of 133.66 Gb of clean data was obtained, yielding 252,342 transcripts and 204,683 unigenes. Differential expression analysis revealed 9878 significantly differentially expressed genes (DEGs) between age groups, with 4252 up-regulated and 5626 down-regulated genes in Y1 compared to Y0. Functional enrichment analysis identified key pathways, including ECM–receptor interaction, PI3K-Akt signaling, WNT signaling, and TGF-β signaling pathways. Notable findings included up-regulation of keratin genes (*KRT1*) and WNT family genes (*WNT3*, *WNT4*, *WNT5*, *WNT6*, *WNT7*, *WNT10*) in one-year-old foals, while collagen genes (*COL1A*, *COL4A*, *COL5AS*) and TGF-β signaling components (*TGFB2*, *TGFB3*, *BMP5*) were down-regulated. These results suggest that skin maturation involves enhanced barrier function, hair follicle development, and reduced collagen synthesis rates, providing insights into mammalian skin development mechanisms and potential applications in veterinary medicine and comparative biology.

## Simple Summary

This study characterized molecular changes during early postnatal skin development in Dezhou donkeys by comparing transcriptomic profiles between newborn and yearling foals. RNA sequencing of skin samples from 13 animals revealed extensive gene expression remodeling during the first year of life, with predominant down-regulation of genes associated with rapid tissue construction and matrix deposition. The molecular signature indicated a developmental transition from active structural formation to maintenance-focused homeostasis. Enhanced expression of barrier-related genes and hair follicle development pathways in yearlings suggests continued skin maturation well beyond birth. These findings provide molecular insights into age-dependent skin characteristics that could inform management practices and quality assessment strategies in donkey production systems.

## 1. Introduction

The domestic donkey (*Equus asinus*) represents one of the most important working animals worldwide, serving diverse roles in agricultural systems, transportation, and livestock production across developing and developed nations [1,2]. Beyond their traditional role as working animals, donkeys have gained significant recognition for their valuable biological products that contribute to human welfare and economic development [3]. Donkey milk, renowned for its nutritional composition and therapeutic properties, closely resembles human breast milk in terms of protein content, lactose levels, and immunological factors, making it particularly valuable for infant nutrition and cosmetic applications [4,5,6]. The meat from donkeys provides a lean protein source with favorable amino acid profiles and is consumed in various cultures, particularly in Mediterranean and Asian regions [7,8]. Perhaps most notably, donkey skin has emerged as a highly valued commodity in traditional medicine and modern industries [9]. It serves as the primary raw material for producing ejiao, a traditional Chinese medicine gelatin with purported health benefits. This has led to unprecedented global demand. The surge in demand has significant economic implications for donkey farming systems [10,11].

Understanding the molecular mechanisms underlying skin development and maturation in donkeys has become increasingly critical, given the growing commercial importance of donkey skin products and the need for sustainable production practices. Skin tissue transcriptomics provides powerful insights into the complex gene expression patterns that govern skin development, cellular differentiation, collagen synthesis, and tissue maturation processes [12,13,14]. The comparison of transcriptomic profiles between different developmental stages offers a unique opportunity to identify age-specific molecular signatures, developmental pathways, and regulatory networks that influence skin quality and characteristics. In donkeys, the skin undergoes significant structural and biochemical changes during the transition from neonatal to juvenile stages, including alterations in collagen composition, dermal thickness, hair follicle development, and extracellular matrix organization [15]. These age-related changes directly impact the quality and commercial value of skin-derived products, making it essential to understand the underlying molecular mechanisms. Furthermore, transcriptomic analysis at different developmental stages can reveal critical genes and pathways involved in skin maturation, potentially identifying biomarkers for skin quality assessment and informing breeding strategies for enhanced skin production traits. The application of RNA-Seq technology enables comprehensive profiling of the skin transcriptome, facilitating the discovery of novel genes, regulatory elements, and biological pathways that may be targeted for improving donkey skin quality and production efficiency [16]. Despite the economic and scientific importance of donkey skin, there remains a significant knowledge gap regarding the molecular basis of age-related skin development in this species. Therefore, in this study, we performed comparative transcriptomic analysis of skin tissues from newborn and one-year-old Dezhou donkey foals using RNA-sequencing technology to characterize the molecular changes occurring during early postnatal skin development. We identified differentially expressed genes between these developmental stages and conducted functional enrichment analysis to reveal the key biological pathways involved in skin maturation, including ECM–receptor interaction, WNT signaling, TGF-β signaling, and PI3K-Akt signaling pathways. Additionally, we constructed protein–protein interaction networks to identify hub genes and elucidate potential regulatory mechanisms underlying the transition from neonatal structural development to mature skin homeostasis.

## 2. Materials and Methods

### 2.1. Ethical Statement

Skin tissue specimens were collected from donkey carcasses at predetermined anatomical sites following standardized veterinary sampling protocols. All animal procedures and experimental protocols were conducted in accordance with the guidelines for the care and use of laboratory animals and were approved by Liaocheng University’s Animal Care and Ethics Committee (Approval No. 2023042602). The research was performed in compliance with institutional guidelines and relevant national regulations for animal welfare.

### 2.2. Sample Collection, Study Design

The study population consisted of 13 Dezhou donkeys stratified into two distinct age cohorts: seven neonatal animals (0–1 days of age; n = 7: 3 males, 4 females) and six yearling animals (12–15 months of age; n = 6: 3 males, 3 females). All 13 animals originated from the same production facility. Samples from the neonatal group were obtained from individuals who died of natural perinatal causes within 24 h of birth, with deaths unrelated to infectious disease processes. All samples were collected directly from the production farm. All animals in the one-year-old group were healthy individuals randomly selected from a population meeting the specified age requirement. Prior to processing, donkeys were fasted for 12 h with free access to water. Animals were transported to a commercial slaughterhouse (Dong’e Tianlong Food Co., Ltd., Liaocheng, China) for meat production, where they were humanely stunned and exsanguinated according to standard commercial practices. Skin tissue specimens for research purposes were collected from the carcasses immediately following hide removal. Dorsal skin samples were excised using a dermal punch. To minimize potential regional variation in gene expression within dorsal skin, all tissue samples were collected from standardized anatomical locations on the left side of the dorsal midline. Specifically, the samples were taken from the area between the shoulder and the hip, approximately at the mid-trunk level corresponding to the 12th–14th thoracolumbar vertebrae. This consistent sampling approach was implemented to reduce confounding effects from regional heterogeneity in skin gene expression, immediately preserved in liquid nitrogen, and subsequently stored at −80 °C for transcriptomic analysis. To minimize variations caused by sampling environments, all samples from the 1-year-old group were processed within a standardized slaughterhouse, while all samples from the 0-year-old group were processed in the farm veterinary workroom. Individual animals were systematically identified using a standardized nomenclature system where “Y” denoted age in years, “F” indicated female gender, and “M” indicated male gender.

### 2.3. RNA Extraction

Total RNA was extracted using the TRIzol method following established protocols. Briefly, tissue samples were thoroughly homogenized in liquid nitrogen and transferred to pre-chilled 1.5 mL centrifuge tubes containing 1 mL TRIzol reagent. Samples were vigorously mixed to ensure complete tissue lysis and incubated at room temperature for 5 min. For tissues requiring extended extraction, this incubation period was appropriately extended. Following initial incubation, samples were centrifuged at 13,000× *g* for 5 min at 4 °C. The upper aqueous phase was carefully transferred to a new centrifuge tube, and 200 μL of pre-chilled chloroform (0.2 mL chloroform per 1 mL TRIzol) was added. Samples were mixed thoroughly and incubated at room temperature for 5 min. After centrifugation at 13,000× *g* for 15 min at 4 °C, approximately 400 μL of the upper aqueous phase was transferred to a new centrifuge tube, avoiding contact with the intermediate protein layer. An equal volume of prechilled isopropanol was added, and samples were incubated at room temperature for 10 min to precipitate RNA. Following centrifugation at 13,000× *g* for 10 min at 4 °C, the supernatant was discarded, revealing a small white pellet at the tube bottom (which may not be visible when RNA quantities are minimal). The pellet was washed with 1 mL of pre-chilled 75% ethanol to remove residual salts. After centrifugation at 12,000× *g* for 5 min at 4 °C, the supernatant was carefully removed using 1000 μL pipette tips, followed by brief centrifugation to collect residual liquid. Remaining liquid was removed using 10 μL pipette tips, and the pellet was air-dried at room temperature for 3–5 min. Care was taken to avoid over-drying, which could render RNA insoluble and promote degradation. Finally, RNA pellets were resuspended in 20–50 μL of sterile 0.1% DEPC-treated water, with the volume adjusted according to the expected RNA yield.

### 2.4. Library Construction and Sequencing

Eukaryotic mRNA sequencing was performed using the Illumina platform to sequence all mRNA transcripts from specific tissues during defined temporal periods. Library construction employed the Illumina TruSeq™ RNA Sample Prep Kit (Illumina, San Diego, CA, USA) following the manufacturer’s protocol. Total RNA concentration and purity were determined using a NanoDrop 2000 spectrophotometer (Thermo Scientific, Wilmington, NC, USA). RNA integrity was assessed by agarose gel electrophoresis, and RNA Integrity Number (RIN) values were determined using an Agilent 5300 system (Agilent Technologies, Palo Alto, CA, USA). Quality criteria for library construction required a total RNA amount ≥ 1 μg, concentration ≥ 30 ng/μL, RIN > 6.5, and OD_260_/_280_ ratios between 1.8 and 2.2. Poly(A)+ mRNA was enriched using oligo(dT) magnetic beads, exploiting the poly(A) tail structure characteristic of eukaryotic mRNA 3′ ends. The A-T base pairing between the poly(A) tail and oligo(dT)-conjugated magnetic beads enabled separation of mRNA from total RNA for subsequent transcriptome analysis. Since the Illumina platform is optimized for short sequence fragments, while enriched mRNA represents complete sequences averaging several kilobases in length, random fragmentation was necessary. Using appropriate fragmentation buffer under optimized conditions, mRNA was randomly cleaved into fragments of approximately 300 base pairs. Single-strand cDNA was synthesized from mRNA templates using random primers and reverse transcriptase, followed by second-strand synthesis to generate stable double-stranded cDNA structures. Double-stranded cDNA with sticky ends was processed using End Repair Mix (Thermo Fisher Scientific, Waltham, MA, USA) to generate blunt ends. Subsequently, adenine bases were added to the 3′ ends to facilitate ligation with Y-shaped adapters. Adapter-ligated products were purified and subjected to fragment size selection. Selected fragments were amplified by PCR to generate the final sequencing library.

### 2.5. Sequencing Data Analysis

Libraries were quantified using Qubit 4.0 (Thermo Scientific, Wilmington, NC, USA) fluorometry and pooled in appropriate ratios for sequencing. Cluster generation was performed via bridge PCR amplification on the cBot system. Final sequencing was conducted on the Illumina NovaSeq 6000 platform following standard protocols. After quality control, high-quality clean reads were subjected to de novo transcriptome assembly using Trinity software (v2.8.5) with default parameters. Assembly quality was evaluated using TransRate (v1.0.3) and BUSCO (v5.2.2) to assess completeness and accuracy. Read mapping was performed using Bowtie2 (v2.3.5), and gene expression levels were quantified using RSEM (RNA-Seq by Expectation-Maximization) with FPKM (Fragments Per Kilobase of transcript per Million mapped reads) normalization. Differential expression analysis between Y0 and Y1 groups was conducted using DESeq2 (v1.30.0) with the following criteria: |log2FC| ≥ 1 and adjusted *p*-value (padj) < 0.05 using the Benjamini–Hochberg method for false discovery rate correction. Sequencing was performed using 150 bp paired-end reads.

### 2.6. Functional Enrichment Analysis and Differentially Expressed Genes (DEGs)

Due to the lack of a reference genome, reads were assembled into unigenes, which were then used as reference for differential expression analysis to identify DEGs. The assembled unigenes were functionally annotated by alignment against multiple databases, including NCBI Nr (Non-redundant protein database), Swiss-Prot, Gene Ontology (GO), KEGG (Kyoto Encyclopedia of Genes and Genomes), Pfam, and KOG/COG. BLASTX (v2.14.1) searches were performed against the NCBI Nr database with an E-value threshold of 1 × 10^−5^. Gene names were assigned based on the best BLAST hit with the highest sequence similarity and lowest E-value. Annotations were cross-referenced with Swiss-Prot and species-specific databases to confirm gene identity. While unigenes were annotated using six different databases (Nr, Swiss-Prot, GO, KEGG, Pfam, and KOG/COG) to ensure comprehensive functional characterization, subsequent enrichment analyses focused specifically on GO and KEGG databases. This selection was based on the fact that GO and KEGG provide the most comprehensive frameworks for understanding biological processes, molecular functions, and metabolic pathways relevant to developmental biology. Each assembled unigene received annotations from all databases where significant matches were identified. GO enrichment analysis examined overrepresented biological processes, molecular functions, and cellular components among DEGs, while KEGG enrichment analysis identified significantly affected metabolic and signaling pathways. These two complementary approaches together provide both broad functional categorization (GO) and specific pathway-level insights (KEGG) into skin development processes.

To determine the biological significance of DEGs, functional enrichment analysis was performed. GO analysis utilized the GO database [17] to classify DEGs according to their associated molecular functions, cellular components, and biological processes. All identified DEGs were mapped to corresponding GO terms through comparison with the Gene Ontology database (http://www.geneontology.org/). Pathway enrichment analysis was carried out using KEGG (Kyoto Encyclopedia of Genes and Genomes) annotation (http://www.genome.jp/kegg (accessed on 14 December 2024)) [18] to examine the involvement of DEGs in biological pathways. Statistical significance was assessed using false discovery rate (FDR) correction through the Benjamini–Hochberg method. GO terms and KEGG pathways with *p* values less than 0.05 were considered statistically significant.

### 2.7. Protein–Protein Interaction (PPI) Network Construction

The top ten up- and down-regulated differentially expressed genes, together with DEGs residing in skin-development-related pathways, were submitted to the STRING database, selecting high-confidence horse interactions (score ≥ 0.7) for protein–protein interaction (PPI) network construction. The resulting PPI relationships were then imported into Cytoscape 3.9.1, subjected to betweenness centrality calculation and ranking, and subsequently exported for further analysis.

## 3. Results

### 3.1. Sequence Quality Control

The sequencing quality control results demonstrate excellent data quality and sufficient coverage for downstream analysis, with a substantial total of 133.66 Gb of high-quality clean data generated, and each individual sample achieving a minimum of 6.14 Gb of clean data, representing robust sequencing depth (Table 1). The initial sequencing produced over 43.03 million raw reads per sample group, and following quality filtering to remove low-quality sequences and poly-N artifacts, more than 41.19 million clean reads were retained per group, indicating minimal data loss during quality control with a retention rate exceeding 95%. Several key quality indicators confirm the high standard of the sequencing data, including extremely low error rates ranging from 0.02% to 0.03% across all sample groups, indicating highly accurate base calling; balanced nucleotide composition with GC percentages between 47.86% and 52.34% falling within expected ranges for most biological samples; and Q30 scores above 92.06% for all sample groups, meaning that over 92% of bases have a base-calling accuracy of 99.9% or higher. These metrics collectively indicate that the sequencing data meets high-quality standards and is suitable for reliable downstream bioinformatics analyses, including variant calling, differential expression analysis, or other genomic investigations.

### 3.2. Transcriptome Assembly Quality Assessment

**Figure 1:**
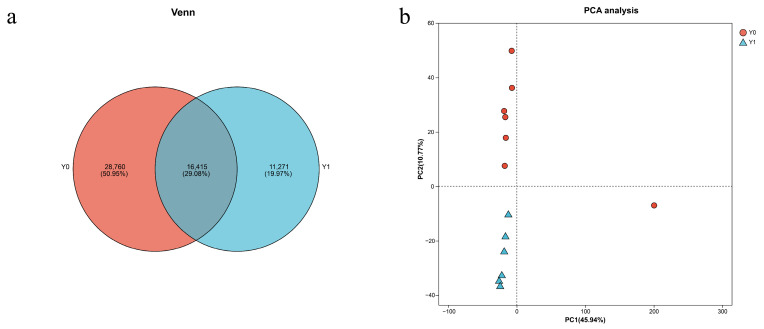
(**a**) Venn diagram between samples; (**b**) Inter-sample PCA analysis.

The assembly results yielded a total of 252,342 transcripts across all samples, with an average length of 1195.59 bp and an N50 length of 2745 bp. The analysis identified 204,683 unigenes with lengths ranging from 201 bp to 23,438 bp, demonstrating an average length of 879.09 bp and an N50 length of 1643 bp (Table 2). The Venn diagram analysis (Figure 1a) illustrates the distribution of expressed genes across different samples, showing substantial overlap in gene expression profiles between age groups while also revealing age-specific transcripts. Principal component analysis (PCA, Figure 1b) demonstrates clear separation between newborn (Y0) and one-year-old (Y1) groups along the first principal component, indicating substantial transcriptomic differences between these developmental stages. The tight clustering of samples within each age group confirms biological reproducibility and validates our experimental design. One Y0 sample shows slightly greater variation (Y0-6M), which may reflect individual biological variation or the heterogeneity inherent in neonatal samples, but remains within acceptable limits for inclusion in downstream analyses.

### 3.3. Functional Annotation Analysis

**Figure 2:**
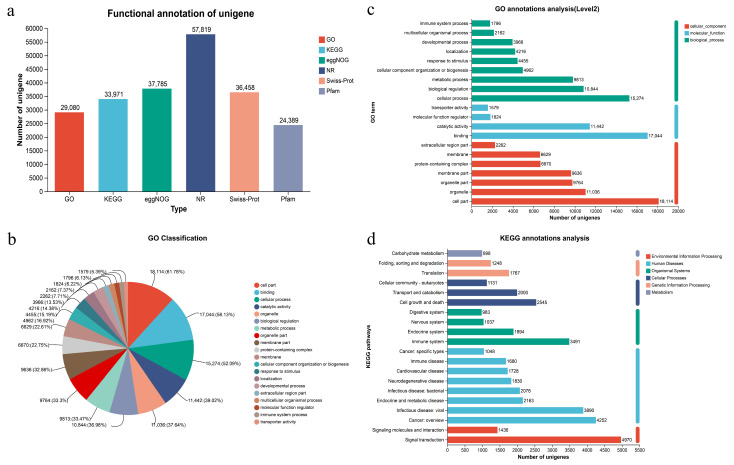
(**a**) Annotated column chart of unigenes in 6 databases at different ages of donkeys; (**b**) Fan chart of unigenes’ GO function; (**c**) Functional annotation histogram of unigenes GO; (**d**) Notes on KEGG functions of unigenes.

Functional annotation of the transcriptome data was performed using six databases for a total of 201,918 expressed unigenes. Of these, 29,080 were annotated in GO, 33,971 in KEGG, 37,785 in eggNOG, 57,819 in NR, 36,458 in Swiss-Prot and 24,389 in Pfam. Overall, 62,837 unigenes (31.12%) acquired at least one functional assignment (Figure 2a). GO analysis, utilizing a standardized classification system that categorizes genes and gene products into three functional domains (molecular function, cellular component, and biological process), annotated 29,080 unigenes, representing 14.4% of all unigenes. Within molecular functions, “binding” (17,044 unigenes; 58.61%) and “catalytic activity” (11,442 unigenes; 39.34%) were the predominant categories. For cellular components, the most abundant categories were “cell part” (18,114 unigenes; 62.29%), “organelle” (11,036 unigenes; 37.95%), “organelle part” (9764 unigenes; 33.13%), “membrane part” (9636 unigenes; 33.13%), “protein-containing complex” (6670 unigenes; 22.93%), and “membrane” (6629 unigenes; 22.79%). In biological processes, “cellular process” (15,274 unigenes; 52.52%), “biological regulation” (10,844 unigenes; 37.29%), and “metabolic process” (9813 unigenes; 33.74%) were the most prominent functional categories (Figure 2b,c). The KEGG pathway annotation identified 33,971 genes distributed across 44 secondary categories within six primary functional classifications, with notable enrichment in signal transduction, cancer overview, viral infectious diseases, immune system, cell growth and death, endocrine and metabolic diseases, bacterial infectious diseases, and transport and catabolism pathways (Figure 2d).

### 3.4. Differentially Expressed Genes

**Figure 3:**
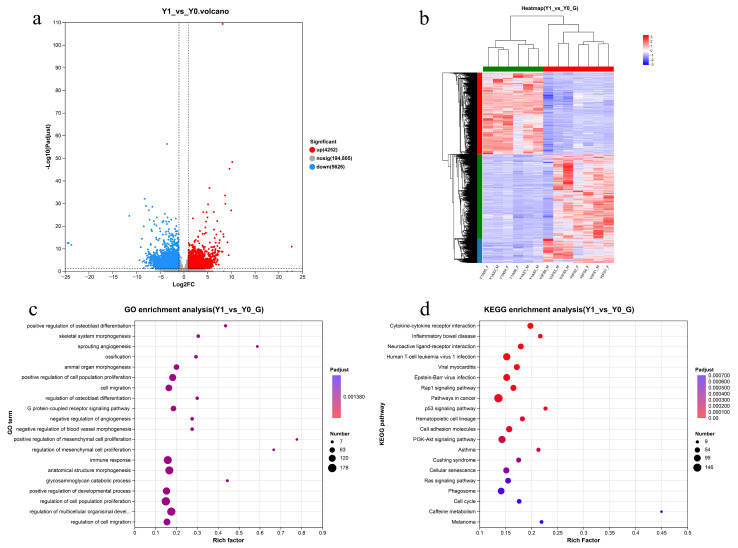
(**a**) DEGs volcano maps for different age groups; (**b**) Heat maps for cluster analysis; (**c**) GO function analysis of DEGs; (**d**) KEGG functional analysis of DEGs. Note: The letter G at the end of the titles for Figures (**c**,**d**) denotes gene set data.

Differential expression analysis between Y1 and Y0 samples revealed 9878 DEGs (Padjust < 0.05), comprising 4252 up-regulated and 5626 down-regulated genes in Y1 relative to Y0 samples, as illustrated in the volcano plot (Figure 3a). Hierarchical clustering of the differentially expressed genes clearly separated the newborn and one-year-old samples into two major branches (Figure 3b).Gene Ontology analysis of the DEGs identified 52 significant GO terms, with the most abundant annotations being cell part (1781 genes), membrane part (1033 genes), organelle (1001 genes), binding (1663 genes), catalytic activity (953 genes), cellular process (1513 genes), and biological regulation (1217 genes), as shown in Figure 3c. KEGG enrichment analysis of DEGs between the age groups demonstrated significant enrichment across 43 secondary pathway categories (Figure 3d), with the most prominently enriched pathways including signal transduction, viral infectious diseases, cancer overview, immune system, cell growth and death, signaling molecules and interaction, endocrine and metabolic diseases, and endocrine system pathways. In Table 3, we list the ten DEGs with the largest fold changes, including both up- and down-regulated genes.

### 3.5. Functional Enrichment Analysis of DEGs

The GO and KEGG pathway enrichment analyses were conducted on DEGs between Y1 and Y0 groups, with the top 20 most significantly enriched pathways and biological processes selected for each analysis, respectively. KEGG enrichment analysis revealed significant enrichment in systemic lupus erythematosus (*p* = 5.86 × 10^−6^, n = 73), PI3K-Akt signaling pathway (*p* = 5.86 × 10^−6^, n = 105), and ECM–receptor interaction (*p* = 6.40 × 10^−3^, n = 33), as demonstrated in Figure 3d. GO enrichment analysis identified significant enrichment in positive regulation of osteoblast differentiation (*p* = 3.58 × 10^−7^, n = 17), animal organ morphogenesis (*p* = 2.26 × 10^−6^, n = 73), and negative regulation of angiogenesis (*p* = 2.72 × 10^−6^, n = 24), as shown in Figure 3c. To identify genes associated with skin development, skin development-related pathways were specifically selected from the KEGG analysis, including ECM–receptor interaction, PI3K-Akt signaling pathway, Wnt signaling pathway, MAPK signaling pathway, and TGF-beta signaling pathway. Although the TGF-beta signaling pathway yielded a *p*-value of 0.07 and did not reach the conventional threshold for statistical significance, it exhibited a trend toward significance, and we therefore included it in Table 4.

### 3.6. PPI Network Analysis of DEGs

**Figure 4:**
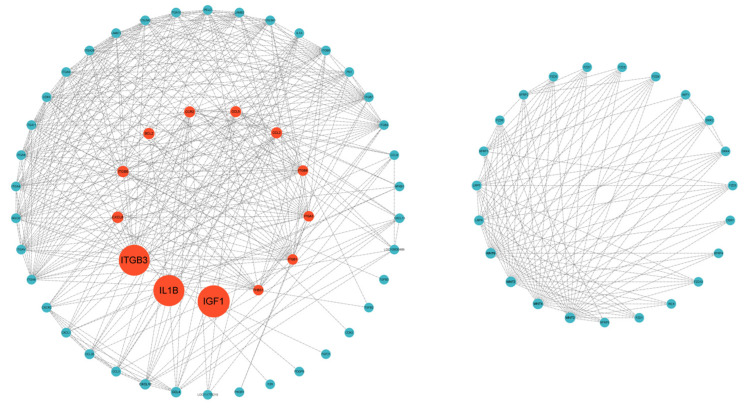
STRING protein–protein interaction analysis.

To systematically dissect the functional landscape of differentially expressed genes (DEGs) implicated in dermal morphogenesis and to uncover potential functional inter-relationships, the top ten up- and down-regulated DEGs, together with DEGs residing in skin-development-related pathways, were submitted to the STRING database for protein–protein interaction (PPI) network construction (Figure 4). The resultant interactome revealed a cohort of pivotal structural regulators, notably IGF1, IL1B, ITGB3, BCL2, CCR2, THBS1, WNT3 and WNT4. Among these, IGF1 and IL1B exhibited the highest degree of connectivity, underscoring their central roles in epidermal proliferation and immune maturation. Intriguingly, robust edges were detected between members of the WNT and FZD families, implying a tightly coupled ligand–receptor axis that may critically orchestrate cutaneous development.

## 4. Discussion

This study provides the first comprehensive transcriptomic analysis of skin development in donkeys during the critical transition from neonatal to juvenile stages. The identification of 9878 DEGs between newborn and one-year-old donkeys reveals significant molecular remodeling occurring during early postnatal skin maturation. The predominance of down-regulated genes (5626 vs. 4252 up-regulated) in one-year-old animals suggests a shift from active developmental processes toward homeostatic maintenance, consistent with the establishment of mature skin architecture and function. Specifically, these results supported our hypothesis by demonstrating that: (1) structural development transitions to maintenance phase through down-regulation of collagen and ECM genes; (2) barrier function matures through up-regulation of *KRT1* and keratinization pathways; and (3) hair follicle cycling is established through coordinated WNT signaling activation.

The significant enrichment of the ECM–receptor interaction pathway, coupled with widespread down-regulation of collagen genes (*COL1A*, *COL4A*, *COL5A*, *COL6A1*, *COL6A3*, *COL28A*) and matrix-associated genes (*THBS1*, *FN1*), indicates substantial extracellular matrix remodeling during the first year of life. This pattern reflects the transition from the rapid matrix deposition characteristic of neonatal skin development to the more stable matrix composition of mature skin [19,20]. Thrombospondin-1 (*THBS1*) down-regulation is particularly significant, as this matricellular protein plays crucial roles in angiogenesis regulation, cell migration, and matrix assembly during development [21,22]. The concurrent down-regulation of fibronectin (*FN1*), a key scaffolding protein for matrix organization, suggests that the initial framework for skin architecture has been established by one year of age. These findings align with previous studies in other mammals showing that ECM composition undergoes substantial reorganization during postnatal development, with a shift from provisional matrix proteins toward more stable structural components [23]. The reduced expression of collagen genes, while initially counterintuitive, likely reflects the completion of initial dermal architecture establishment. In mature skin, collagen turnover occurs at much lower rates than during active development, explaining the observed down-regulation. This interpretation is supported by the concurrent reduction in matrix metalloproteinase activity-related pathways, suggesting achievement of matrix homeostasis.

The significant up-regulation of *KRT1* in one-year-old donkeys indicates enhanced epidermal barrier function and mechanical resistance. The *KRT1*, a major component of the cornified envelope in stratified epithelia, is essential for skin barrier integrity and protection against environmental stressors [24,25]. This up-regulation reflects the adaptation to increased environmental challenges faced by growing animals, including temperature fluctuations, UV exposure, and mechanical stress. The enhanced keratin expression aligns with the observed enrichment of biological processes related to epidermal development and keratinization. This molecular signature suggests that while overall skin development has progressed significantly by one year, the epidermal barrier continues to strengthen in response to environmental demands. The evolutionary adaptation of enhanced keratin expression in juvenile animals likely provides survival advantages in diverse environmental conditions.

The significant up-regulation of multiple WNT family members (*WNT3*, *WNT4*, *WNT5*, *WNT6*, *WNT7*, *WNT10*) and BMP3 provides strong evidence for active hair follicle development and cycling regulation in one-year-old donkeys. The WNT/β-catenin signaling pathway is fundamental to hair follicle morphogenesis, stem cell maintenance, and cycling regulation [26,27,28]. The observed up-regulation likely reflects the establishment of mature hair follicle cycling patterns and the development of the adult hair coat. The enrichment of WNT signaling is consistent with the transition from fine neonatal hair to the coarser, more protective adult coat observed in donkeys. This molecular signature suggests that hair follicle maturation extends well beyond birth, with significant developmental processes continuing through the first year of life. The coordinate regulation of multiple WNT family members indicates the complexity of hair follicle development and the need for precise spatiotemporal control of signaling gradients [29].

The down-regulation of TGF-β family members (*TGFB2*, *TGFB3*) and *BMP5* suggests important changes in growth factor signaling networks during skin maturation. TGF-β signaling plays crucial roles in cell proliferation, differentiation, and matrix production [30]. The reduced expression of *TGFB2* and *TGFB3* in older animals may contribute to the decreased propensity for excessive scar formation observed in young mammals, as neonatal skin typically exhibits enhanced healing with minimal scarring. The down-regulation of *BMP5*, which is essential for mechanoreceptor development and sensory function [31,32], suggests that the initial establishment of cutaneous sensory networks is largely complete by one year of age. This finding has important implications for understanding the critical periods of sensory system development in donkeys and may inform management practices for young animals.

The significant enrichment of cytokine–cytokine receptor interaction pathways reflects the maturation of skin immune functions during early postnatal development. The differential expression of cytokine signaling components *(CCL4*, *CCR2*, *CXCR2*, *IL1A*) indicates establishment of immunological competence in maturing skin, which is essential for barrier defense function. The enrichment of p53 signaling pathway (*CDKN2A*, *ATR*, *IGF1*, *BCL2*, *CDK2*, *MDM4*) suggests active regulation of cell cycle control and apoptosis during tissue remodeling. The p53 pathway plays crucial roles in maintaining genomic stability during rapid cell division and differentiation, which are characteristic of developing tissues. Together, these pathways indicate that skin maturation involves not only structural changes but also establishment of regulatory mechanisms governing cellular homeostasis and immune defense capabilities.

The enrichment of PI3K-Akt signaling pathway reflects changes in cellular metabolism, survival, and growth regulation during skin maturation. This pathway integrates multiple growth signals and regulates cell proliferation, differentiation, and survival. The altered expression of PI3K-Akt pathway components suggests a shift from growth-promoting signals in neonatal skin toward maintenance and homeostatic signaling in juvenile animals. The down-regulation of β-catenin (*CTNNB1*), a key component of both Wnt signaling and cell adhesion complexes, indicates structural maturation of intercellular junctions and potentially reduced stem cell activity compared to neonatal skin [33]. This finding suggests that the high regenerative capacity characteristic of neonatal skin begins to decline as animals approach adulthood.

The down-regulation of heat shock proteins *(HSPA1*) in one-year-old donkeys suggests reduced cellular stress and improved cellular homeostasis compared to neonatal animals. Heat shock proteins serve as molecular chaperones and play crucial roles in cellular stress responses [34,35]. The reduced expression may indicate that the skin has achieved greater stability and resistance to environmental stressors through structural maturation rather than relying on stress response mechanisms. This change has important implications for understanding thermal regulation and stress tolerance in developing donkeys. The transition from stress protein-dependent to structure-dependent protection mechanisms represents an important developmental milestone with practical implications for animal husbandry and management.

The transcriptomic changes observed in donkey skin development show both similarities and differences compared to other mammalian species. The general pattern of reduced matrix synthesis and enhanced barrier function is conserved across mammals, suggesting fundamental developmental programs [36,37]. However, the specific expression patterns of certain genes, particularly those related to hair follicle development, may reflect adaptations to the donkey’s semi-arid habitat origins and their thick, protective coat requirements [15]. The coordinate regulation of multiple developmental pathways highlights the integrated nature of skin maturation and suggests that interventions targeting single pathways may have limited effectiveness. Understanding these natural developmental processes is crucial for developing strategies to enhance skin health and function in domestic donkeys.

While this study provides valuable insights into donkey skin development, several limitations should be acknowledged. The relatively small sample size (13 animals) may limit the detection of subtle expression changes, and the cross-sectional design prevents tracking individual developmental trajectories. Additionally, the analysis focused on bulk tissue RNA-seq, which may obscure cell-type-specific changes that could be important for understanding developmental mechanisms [38,39]. The use of reference transcriptomes from related species for annotation may have introduced biases, and donkey-specific transcripts or splice variants may have been missed. A limitation of this study is the absence of histological validation. Tissue samples were processed exclusively for RNA sequencing, precluding immunohistochemistry, in situ hybridization, or H&E staining to confirm protein-level expression and morphological correlates of the transcriptomic changes observed. Importantly, while these limitations affect the depth of mechanistic interpretation and validation of our findings, they do not compromise the validity or reliability of the transcriptomic results presented. The RNA-seq data processing followed rigorous quality control standards, and the statistical analyses employed established methods with appropriate corrections for multiple testing. The core findings regarding differential gene expression patterns between age groups remain robust and provide a reliable foundation for the biological interpretations discussed.

This foundational study opens several important avenues for future research. Longitudinal studies tracking the same animals over time would provide insights into individual variation in developmental timing and the influence of environmental factors. Single-cell RNA sequencing would allow characterization of cell-type-specific developmental programs and identification of novel cell populations involved in skin development. Functional validation of key DEGs through targeted approaches would strengthen the biological interpretation of the findings. Additionally, comparative studies across different donkey breeds could reveal genetic factors influencing skin development and adaptation to different environments. Investigation of the molecular mechanisms underlying the enhanced wound healing capacity of young donkeys could have important translational applications for both veterinary and human medicine [40]. The development of age-specific management strategies based on the developmental stage of skin barrier function could improve animal welfare and health outcomes.

This transcriptomic analysis reveals that donkey skin undergoes substantial molecular remodeling during the first year of life, with distinct patterns of gene expression reflecting the transition from developmental to homeostatic states. The coordinated regulation of multiple signaling pathways (ECM–receptor interaction, WNT, TGF-β, PI3K-Akt) demonstrates the integrated nature of skin maturation processes. The findings have important implications for donkey husbandry and management, suggesting that animals younger than one year may require different care considerations due to ongoing skin development and potentially different environmental sensitivities [41]. Understanding these natural developmental processes provides a foundation for developing age-appropriate management strategies and may inform approaches to enhance skin health and function in domestic donkeys. From a broader biological perspective, this study contributes to our understanding of mammalian skin development and highlights both conserved and species-specific aspects of cutaneous biology [42,43]. The comprehensive transcriptomic dataset generated provides a valuable resource for future comparative and functional studies in equine biology and mammalian development.

## 5. Conclusions

These results suggest that the up-regulation of Wnt-signaling components (*WNT3*, *WNT4*, *WNT5*, *WNT6*, *WNT7*, *WNT10*) drives skin maturation in one-year-old foals, leading to an enhanced barrier function and active hair follicle development. Conversely, down-regulation of collagen genes (*COL1A*, *COL4A*, *COL5AS*) and TGF-β signaling components (*TGFB2*, *TGFB3*, *BMP5*) suggests a transition from rapid structural development to maintenance phase, indicating that initial skin architecture formation is largely complete by one year of age. The enrichment in immune-related pathways demonstrates maturation of skin immune functions. These findings provide valuable insights into mammalian skin development mechanisms and establish molecular signatures that could serve as biomarkers for developmental assessment in veterinary medicine and breeding programs. Future studies should incorporate additional developmental time points to better understand the temporal dynamics of skin maturation in equids. Furthermore, future investigations should include histological analyses (such as immunohistochemistry or in situ hybridization) and molecular validation techniques (such as RT-qPCR or Western blotting) to confirm protein-level expression of the key genes identified through RNA-seq. Taken together, our findings unveil a previously unknown molecular timeline of postnatal donkey skin maturation that enables precision breeding for enhanced hide yield, early veterinary intervention, and the sustainable exploitation of donkey skin resources. This work represents a significant advance in equine dermatological research, providing the first comprehensive molecular roadmap of postnatal skin development in donkeys and establishing a critical foundation for future studies aimed at improving animal welfare, optimizing breeding programs, and ensuring the sustainability of donkey-derived products in traditional medicine.

## Figures and Tables

**Table 1:** The original data of each sample and the data after quality control.

Sample	Raw Reads	Clean Reads	Q30 (%)	GC Content (%)
Y1-6F	44,718,500	42,617,512	92.75	51.44
Y1-5F	43,961,670	42,224,166	92.79	50.67
Y1-4F	57,855,776	55,420,804	92.52	51.89
Y1-3M	61,201,110	58,631,834	93.40	52.14
Y1-2M	53,822,586	51,608,052	92.62	50.42
Y1-1M	47,547,774	45,434,550	93.16	51.32
Y0-7F	53,427,742	50,980,380	93.08	52.09
Y0-6M	44,209,364	41,983,592	92.23	47.86
Y0-5M	44,369,964	42,410,900	92.70	51.27
Y0-4F	47,127,032	45,161,808	92.75	51.94
Y0-3M	46,000,214	43,668,942	92.06	51.09
Y0-2F	54,753,510	52,625,948	92.95	52.34
Y0-1M	43,030,088	41,192,430	92.91	51.70

**Table 2:** Assembly results evaluation.

Type	Unigene	Transcript
Total number	204,683	252,342
Total base	179,934,544	301,698,306
Largest length (bp)	23,438	23,438
Smallest length (bp)	201	201
Average length (bp)	879.09	1195.59
N50 length (bp)	1643	2745
E90N50 length (bp)	5349	4653
Fragment mapped percent (%)	57.336	75.865
GC percent (%)	47.88	48.99
TransRate score	0.31369	0.40984
BUSCO score	C: 74.2% [S: 68.4%; D: 5.8%]	C: 91.2% [S: 49%; D: 42.2%]

**Table 3:** The top 10 significantly upregulated and downregulated DEGs.

Gene ID	Gene Symbol	Description	Gene Location	log2FC	
TRINITY_DN52064_c0_g1	*lapB*	lipopolysaccharide assembly protein B	-	−24.42	5.12377227885 × 10^−16^
TRINITY_DN124302_c0_g1	-	-	-	−24.20	5.768754256059999 × 10^−16^
TRINITY_DN11936_c0_g1	-	-	-	−23.65	4.1698098576900005 × 10^−15^
TRINITY_DN2379_c0_g1	*DLK1*	delta-like non-canonical Notch ligand 1	NC_091796.1 (105426265…105434305)	−11.46	9.046755655739999 × 10^−29^
TRINITY_DN8109_c2_g1	*CB1*	Description: hypothetical protein CB1_000632073	NC_091813.1 (26743405…26770803, complement)	−9.30	2.24600248567 × 10^−7^
TRINITY_DN3088_c0_g4	*VEPH1*	ventricular zone expressed PH domain containing 1	NC_091794.1 (59204382…59416979)	−9.05	5.4593363009899994 × 10^−14^
TRINITY_DN3088_c0_g5	*PTX3*	PREDICTED: pentraxin-related protein PTX3	NC_091794.1 (59262091…59272355, complement)	−8.81	5.5530348186 × 10^−18^
TRINITY_DN4805_c0_g3	*TNFAIP6*	TNF alpha-induced protein 6 isoform X2	NC_091793.1 (93492733…93512732)	−8.65	1.47041866536 × 10^−6^
TRINITY_DN4805_c0_g2	*TNFAIP6*	TNF alpha-induced protein 6	NC_091793.1 (93492733…93512732)	−8.42	7.14542107058 × 10^−24^
TRINITY_DN26180_c0_g1	*KRT1*	keratin, type I cytoskeletal 42-like, partial	NC_091811.1 (54060359…54066039, complement)	−8.42	0.005
TRINITY_DN1796_c0_g4	LOC106833849	boLa class II histocompatibility antigen, DQB*0101 beta chain	NC_091797.1 (31359471…31599679)	22.75	3.11564222773 × 10^−14^
TRINITY_DN10233_c0_g1	*CLCA1*	chloride channel accessory 1	NC_091805.1 (22276421…22304481)	10.23	2.2056893074099997 × 10^−53^
TRINITY_DN58797_c0_g3	LOC106827057	cysteine-rich secretory protein 3	NC_091797.1 (16636067…16660501)	9.99	2.3183481600999997 × 10^−31^
TRINITY_DN8468_c0_g2	*KRT2*	keratin, type II cytoskeletal 2 epidermal-like	NC_091811.1 (54001973…54009543, complement)	9.64	2.9487332673999997 × 10^−50^
TRINITY_DN2330_c0_g1	*IGH*	antigen binding; Immunoglobulin heavy constant epsilon	-	9.46	5.395981605550001 × 10^−10^
TRINITY_DN2795_c0_g1	*KRT2*	Keratin, high-sulfur B2 protein	Chromosome 22, NC_091811.1 (54042239.54048598, complement)	9.22	2.35250771847 × 10^−16^
TRINITY_DN4750_c0_g1	-	PREDICTED: YLP motif-containing protein 1-like	-	8.78	1.7401507674099998 × 10^−34^
TRINITY_DN9228_c0_g3	*KRT2*	keratin, type II cytoskeletal 2 epidermal-like	NC_091811.1 (54001973…54009543, complement)	8.71	2.78253069778 × 10^−38^
TRINITY_DN5354_c0_g1	LOC139039988	keratin-associated protein 4–6-like	NC_091802.1 (40731844…40732741, complement)	8.39	6.208722365599999 × 10^−19^
TRINITY_DN4367_c0_g3	-	-	-	8.36	4.0208659955700004 × 10^−20^

Note: genes are ranked by fold-change magnitude, and all meet the statistical significance threshold of *p*-value < 0.05.

**Table 4:** KEGG pathways associated with skin development.

Pathway ID	KEGG Pathways	DEGs	*p*-Value
map04512	ECM–receptor interaction pathway	*COL1A*, *COL4A*, *COL5A*, *COL6A1*, *COL6A3*, *COL28A*, *LAMC1*, *LAMB2*, *FN1*	0.006
map04151	PI3K-AKT signaling pathway	*FGF*, *IGF1*, *PDGFB*, *F2R*, *IGH*, *PIK3R1_2_3*, *AKT*	5.85880137951 × 10^−6^
map04060	Cytokine–cytokine receptor interaction	*CCL4*, *CCR2*, *CXCR2*, *IL1A*, *TGFB2*, *TGFB3*	4.97307271568 × 10^−10^
map04115	p53 signaling pathway	*CDKN2A*, *ATR*, *IGF1*, *BCL2*, *CDK2*, *MDM4*	1.67009680162 × 10^−6^
map04350	TGF-beta signaling pathway	*BMP5*, *THBS1*, *FBN1*, *THBS1*, *SMAD9*	0.070
map04310	Wnt signaling pathway	*WNT2*, *WNT3*, *WNT4*, *WNT5*, *WNT6*, *WNT7*, *WNT10*, *FZD9*	0.028
map04010	MAPK signaling pathway	*RASGRF1*, *RASGRP2*, *MKP*, *NFKB1*	0.000118318624691

## Data Availability

The original contributions presented in this study are included in the article. Further inquiries can be directed to the corresponding authors.
